# Multiple renal tumorectomy in a Von Hipple Lindau patient. Combined retro/transperitoneal approach with intracorporeal hypotermia

**DOI:** 10.1590/S1677-5538.IBJU.2018.0803

**Published:** 2019-12-17

**Authors:** Valentí Tubau, Jose Luis Bauza, Enrique Pieras, Xavier Brugarolas, Pedro Pizà

**Affiliations:** 1 Department of Urology, Hospital Universitario Son Espases, Palma de Mallorca, Illes Balears, Spain

## Abstract

**Objective & Introduction:**

To show the feasibility of a combined transperitoneal (TP) and retroperitoneal (RP) laparoscopic approach in a Von Hipple-Lindau (VHL) patient with multiple kidney tumors. VHL is an autosomal dominant inherited syndrome characterized by a high incidence of benign and malignant tumors and cysts in many organs. Renal cell carcinoma is one of the most common and a leading cause of mortality (1). Surgical approach is usually complex because of its multiplicity and the need of maximum kidney function preservation due to the risk of future recurrences (2, 3). Intracorporeal renal hypothermia may be useful in these cases to prevent permanent renal function loss (4).

**Materials and Methods:**

A 40 years old male was being monitored for multiple bilateral renal masses. Family history included a VHL syndrome affecting his mother and sister.

Past medical history included a VHL syndrome with multiple cerebellar and medular hemangioblastomas, a pancreatic cystoadenoma and bilateral kidney tumors which had significantly grown up during follow-up.

The patient was scheduled for laparoscopic multiple partial nephrectomy. A combined TP and RP approach with intracorporeal hypothermia was chosen.

**Results:**

A total of six right kidney tumors were removed. Operative time was 240 min. Cold ischemia time was 50 min. Average kidney temperature was 23.7°C. Blood losses were negligible. The patient was discharged after 72 hours. No major changes in serum creatinine were found during the follow-up. Final pathology revealed a clear cell renal cell carcinoma, pT1a, ISUP grade 2 in most of the tumors but one ISUP grade 3. Surgical margins were negative.

**Conclusions:**

Combined TP and RP is a feasible alternative for the treatment of multiple renal tumors. It's safe and effective, allowing the use of intracorporeal hypothermia which may improve postoperative renal function. Consistent experience is needed before embarking on this surgery.

## ARTICLE INFO

**Available at: http://www.intbrazjurol.com.br/video-section/20180803_Tubau_et_al**

**Int Braz J Urol. 2019; 45 (Video #25): 1283-4**

## ABBREVIATIONS

VHL: Von Hipple-Lindau
TP: Transperitoneal
RP: Retroperitoneal
MRI: Magnetic Resonance Image
IVC: Inferior Vena Cava
MAL: Mid Axillary Line
ISUP: International Society of Urological Pathology
